# Vitamin D’s Effect on the Proliferation and Inflammation of Human Intervertebral Disc Cells in Relation to the Functional Vitamin D Receptor Gene FokI Polymorphism

**DOI:** 10.3390/ijms19072002

**Published:** 2018-07-09

**Authors:** Paola De Luca, Laura de Girolamo, Carlotta Perucca Orfei, Marco Viganò, Riccardo Cecchinato, Marco Brayda-Bruno, Alessandra Colombini

**Affiliations:** 1 Orthopaedic Biotechnology Lab, IRCCS Galeazzi Orthopaedic Institute, Via R. Galeazzi 4, 20161 Milan, Italy; deluca.paola@grupposandonato.it (P.D.L.); laura.degirolamo@grupposandonato.it (L.d.G.); carlotta.perucca@grupposandonato.it (C.P.O.); marco.vigano@grupposandonato.it (M.V.); 2 GSpine4, IRCCS Galeazzi Orthopaedic Institute, Via R. Galeazzi 4, 20161 Milan, Italy; dott.cecchinato@gmail.com; 3 Scoliosis Unit, Department of Orthopedics and Traumatology-Spine Surgery III, IRCCS Galeazzi Orthopaedic Institute, Via R. Galeazzi 4, 20161 Milan, Italy; marco.brayda@spinecaregroup.it

**Keywords:** intervertebral disc, vitamin D, vitamin D receptor polymorphism, proliferation, inflammation

## Abstract

Vitamin D is known to have immunomodulatory effects, is involved in osteo-cartilaginous metabolism, and may have a role in human intervertebral disc pathophysiology. Although a link between vitamin D receptor (VDR) gene variants and disc degeneration-related pathologies has been observed, its functional contribution to pathologic processes has not been assessed yet. The aim of this study was to investigate the response of disc cells to vitamin D in terms of the regulation of proliferation, metabolism, and inflammatory processes, with a particular focus on the FokI *VDR* genotype. However, although it was found that vitamin D had a pro-apoptotic effect regardless of genotype, an up-regulation of IL-1Ra and downregulation of IL-6 was found to be evident only in *Ff* cells. Regarding the metabolic effects, in *Ff* cells, vitamin D promoted an upregulation of the aggrecan in inflammatory conditions but did not have an effect on the expression of collagen-related markers. Moreover, cells bearing the *Ff* genotype were the most responsive to vitamin D in the upregulation of catabolic markers. In addition, in contrast to the *FF* genotype, vitamin D downregulated the vitamin D-dependent signaling pathway in inflamed *Ff* cells, counteracting the inflammation-mediated catabolic effects. In conclusion, *Ff* cells were found to be more responsive to the anti-inflammatory and catabolic effects of vitamin D, which is likely to be related to matrix remodeling.

## 1. Introduction

The involvement of the vitamin D endocrine system in the pathophysiology of the human intervertebral disc is still a topic of debate which is not fully explored. Few in-vitro studies have reported that vitamin D regulates proliferation, the expression of matrix genes, production of structural proteins, cytokines, and growth factors in cells obtained from the two main anatomical components of the disc, the nucleus pulposus (NP), and the annulus fibrosus (AF), and expressing the vitamin D receptor (VDR) [1,2]. 

The need to perform functional studies to analyze the effects of vitamin D on the fibro-cartilaginous disc and the osteo-cartilaginous endplate (CEP) resides in a number of evidences describing the association between the four most studied, known genetic *VDR* variants (FokI, BsmI, TaqI and ApaI), and the disc degeneration-related pathologies [3,4], although inconsistent associations have been reported [5,6]. 

Recently, a correlation between the aforementioned genetic variants and specific lumbar spine pathologies, such as herniation, discopathy, and osteochondrosis, has been observed [7,8,9,10]. Some *VDR* alleles and genotypes predisposed to lumbar spine pathologies have been identified in patients with a concomitant increase of type II collagen degradation products, which likely derives from the degradation of the disc’s matrix [11,12]. However, there are still few findings concerning the contribution of these variants to pathologic processes. The FokI polymorphism is particularly interesting for its functional role—in fact, it is located in the start codon of the *VDR* and consists of a C to T transition, determining the transcription of a shorter, allele C (*F* allele), or longer allele T (*f* allele) polypeptide [13]. The shorter polypeptide couples more efficiently with the transcription factor II B than the longer peptides and leads to a higher transcriptional rate of vitamin D-dependent genes [14,15]. 

Moreover, given its involvement in osteo-cartilaginous metabolism, vitamin D might have a crucial role in the degenerative development of the disc and endplate [3]. 

Finally, the immunomodulatory effects of vitamin D have been also suggested [16], albeit without any clear explanation as to its possible involvement in the regulation of the inflammatory and catabolic processes present in the degenerate discs [17,18]. 

Based on this background, the aim of this study was to investigate the in-vitro regulation of proliferation, metabolism, and inflammatory processes of disc cells in response to vitamin D treatment, with a focus on the functional FokI *VDR* genotype. This study attempts to clarify the functional meaning of the association of this genetic variant with the predisposition to the development of disc degeneration-related pathologies.

## 2. Results

### 2.1. Anti-Proliferative Effect of Vitamin D Is Related to Induction of Apoptosis

Vitamin D treatment caused a decrease in the number of viable cells (−2.7%, *p* < 0.01). 

**Figure 1:**
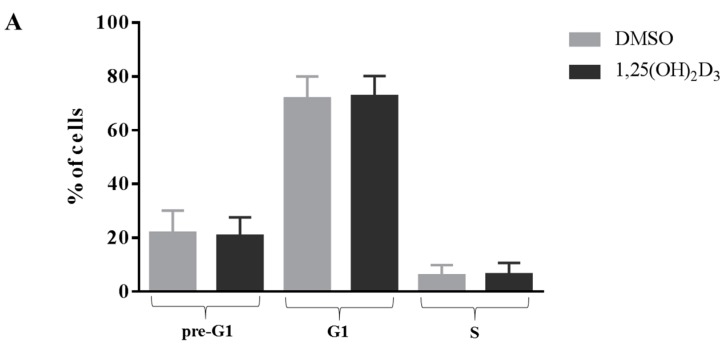

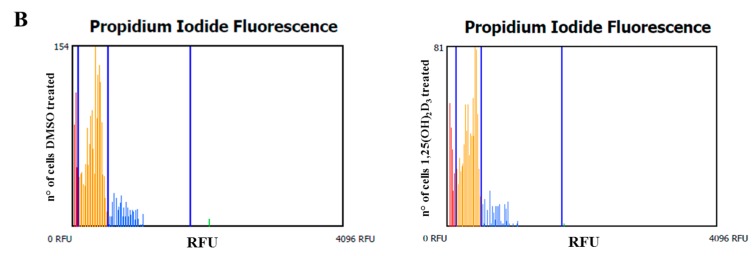
Cell cycle of disc cells in response to vitamin D treatment. (**A**) Shows the percentage of cells bearing both the *FF* and *Ff* genotype in the different cell cycle phases (pre-G1, G1 and S). Light gray and dark gray bars represent the cells treated with DMSO and vitamin D, respectively. Data are represented as mean ± SD, *n* = 15; (**B**) Shows two representative cell cycle’s graphs after DMSO and vitamin D treatment. Red, yellow, blue and green bars represent pre-G1, G1, S and G2-M phases, respectively.

**Figure 2:**
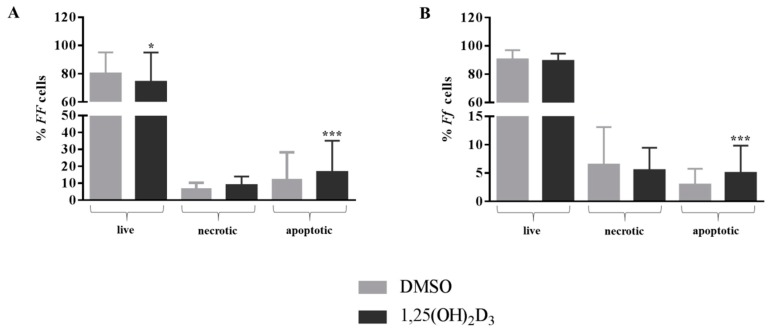
Apoptosis of disc cells in response to vitamin D treatment. Shows percentage of live, necrotic and apoptotic cells bearing *FF* (**A**) and *Ff* (**B**) genotypes. Light and dark gray bars represent the cells treated with DMSO and vitamin D, respectively. * *p* < 0.05, *** *p* < 0.001 vs. DMSO treatment. *FF* genotype *n* = 4, *Ff* genotype *n* = 11. Data are represented as mean ± SD.

The anti-proliferative effect of vitamin D did not affect the cell cycle (Figure 1), but significantly increased the percentage of apoptotic cells (*p* < 0.001). The cells bearing the *FF* genotype showed a slight, but significant, decrease of the number of living cells (−8%, *p* < 0.05) (Figure 2A). However, the rate of apoptosis was significantly increased by the vitamin D treatment in both cell types (+32% and +66%, both *p* < 0.001, for *FF* and *Ff*, respectively) (Figure 2A,B).

### 2.2. Effect of IL-1β on Disc Cells Cultured in Absence of Vitamin D

**Figure 3:**
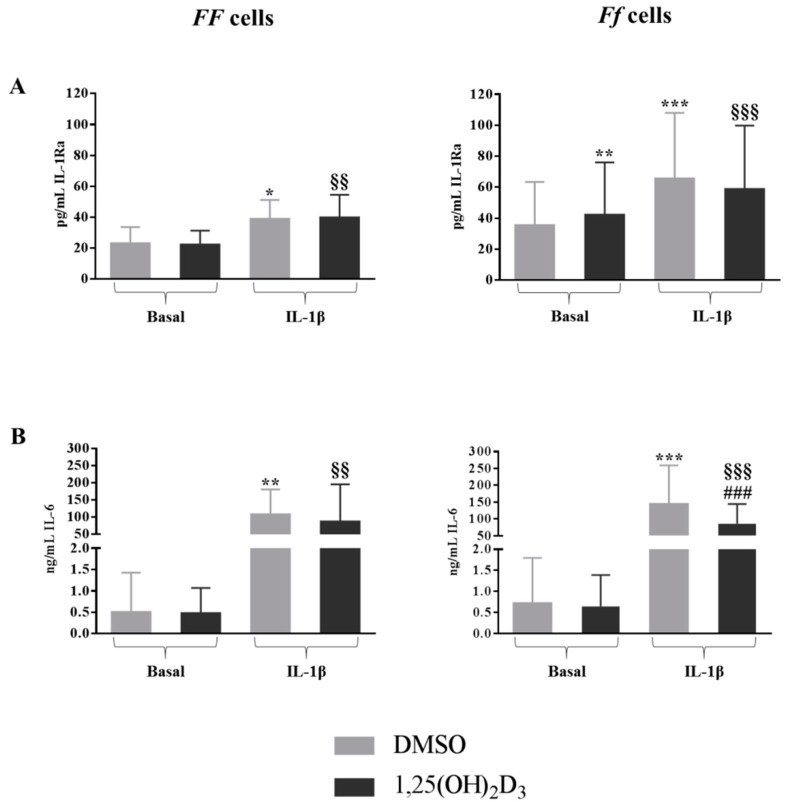
Concentrations of IL1-Ra (**A**) and IL-6 (**B**) released from *FF* and *Ff* bearing cells, in basal and inflamed (IL-1β treatment) conditions, in response to vitamin D treatment. Light and dark gray bars represent the cells treated with DMSO and vitamin D, respectively. * *p* < 0.05, ** *p* < 0.01, *** *p* < 0.001 vs. DMSO treatment in basal condition. ^§§^ *p* < 0.01, ^§§§^ *p* < 0.001 vs. vitamin D treatment in basal condition. ^###^ *p* < 0.001 vs. DMSO + IL-1β treatment. *FF* genotype *n* = 3, *Ff* genotype *n* = 10. Data are represented as mean ± SD.

As expected, IL-1β induced a significantly higher release of IL-1Ra (*p* < 0.05 and *p* < 0.001 for *FF* and *Ff*, respectively) (Figure 3A). Moreover, in cells bearing both the genotypes, the addition of 1 ng/mL IL-1β to the culture provoked a strong increase of the release of IL-6 up to 200-fold (*p* < 0.01 and *p* < 0.001 for *FF* and *Ff*, respectively) (Figure 3B).

**Figure 4:**
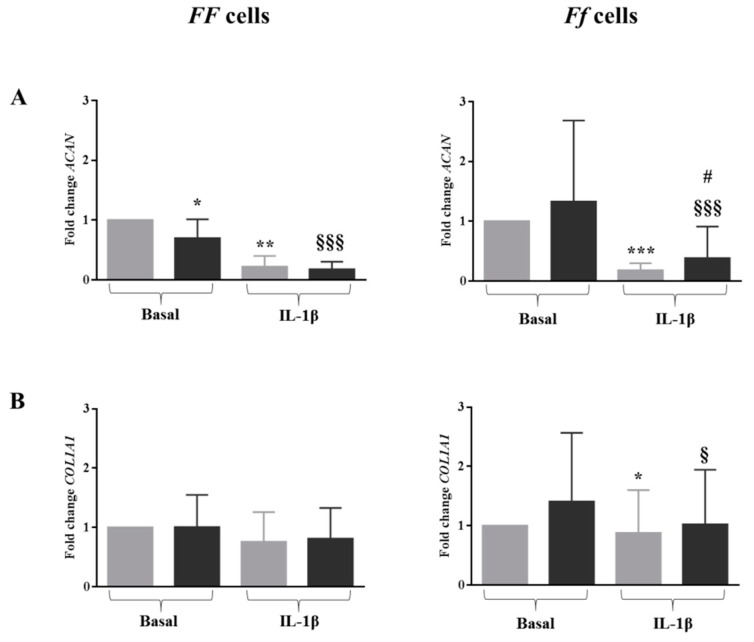

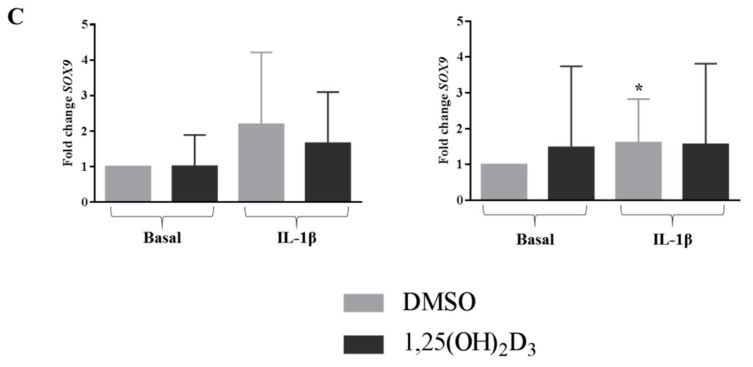
Expression of disc phenotype markers *ACAN* (**A**), *COL1A1* (**B**) and *SOX9* (**C**) in cells bearing *FF* and *Ff* genotypes, in basal and inflamed (IL-1β treatment) conditions, in response to vitamin D treatment. Light and dark gray bars represent the cells treated with DMSO and vitamin D, respectively. * *p* < 0.05, ** *p* < 0.01, *** *p* < 0.001 vs. DMSO treatment in basal condition. ^§^ *p* < 0.05, ^§§§^ *p* < 0.001 vs. vitamin D treatment in basal condition. ^#^ *p* < 0.05 vs. DMSO + IL-1β treatment. *FF* genotype *n* = 3, *Ff* genotype *n* = 7. Data are represented as mean ± SD.

In an inflamed condition, the expression of *ACAN* was strongly downregulated in the cells of both genotypes (*p* < 0.01 and *p* < 0.001 for *FF* and *Ff*, respectively) (Figure 4A). Similarly, the presence of IL-1β slightly decreased the expression of *COL1A1* with respect to the basal culture condition, although it was only in cells bearing the *Ff* genotype, which turned out to be statistically significant (*p* < 0.05) (Figure 4B). Conversely, the expression of *SOX9* was upregulated by IL-1β in cells bearing both genotypes, again only in a significant manner in cells bearing the *Ff* genotype (*p* < 0.05) (Figure 4C). 

**Figure 5:**
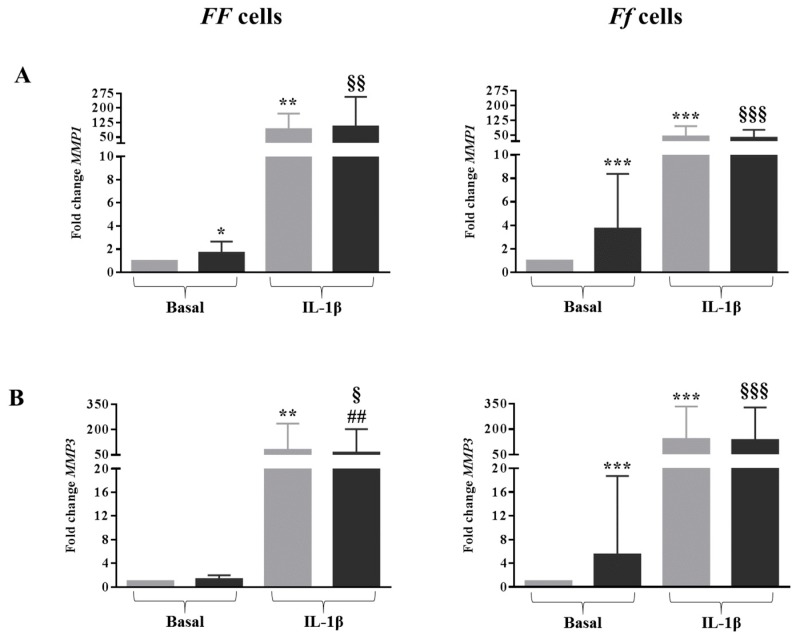

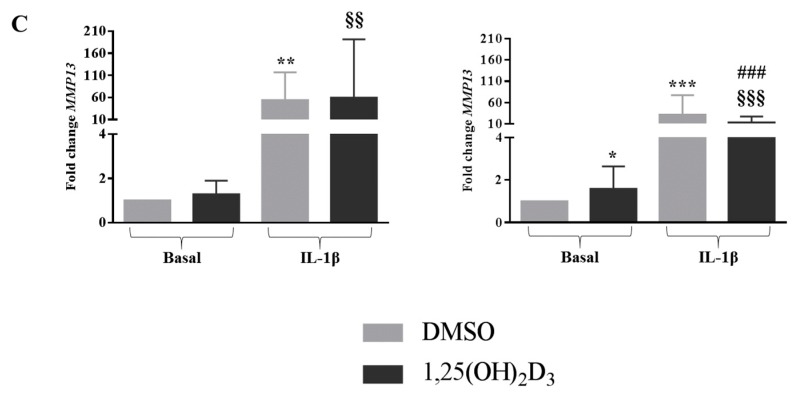
Expression of disc catabolic markers *MMP1* (**A**), *MMP3* (**B**) and *MMP13* (**C**) in cells bearing *FF* and *Ff* genotypes, in basal and inflamed (IL-1β treatment) conditions, in response to vitamin D treatment. Light and dark gray bars represent the cells treated with DMSO and vitamin D, respectively. * *p* < 0.05, ** *p* < 0.01, *** *p* < 0.001 vs. DMSO treatment in basal condition. ^§^ *p* < 0.05, ^§§^ *p* < 0.01, ^§§§^ *p* < 0.001 vs. vitamin D treatment in basal condition. ^###^ *p* < 0.01, ^###^ *p* < 0.001 vs. DMSO + IL-1β treatment. *FF* genotype *n* = 3, *Ff* genotype *n* = 7. Data are represented as mean ± SD.

As expected, IL-1β provoked a very marked increase of *MMP1*, *MMP3* and *MMP13* expression in cells bearing both genotypes (all *p* < 0.01 for *FF* cells and all *p* < 0.001 for *Ff* cells) (Figure 5A–C). 

**Figure 6:**
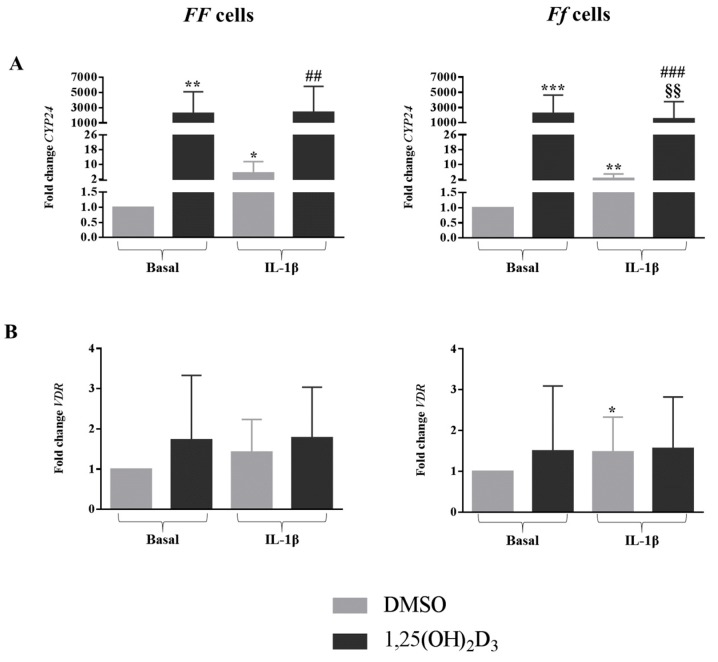
Expression of genes related to vitamin D-dependent signaling pathway *CYP24* (**A**) and *VDR* (**B**), in cells bearing *FF* and *Ff* genotypes, in basal and inflamed (IL-1β treatment) conditions, in response to vitamin D treatment. Light and dark gray bars represent the cells treated with DMSO and vitamin D, respectively. * *p* < 0.05, ** *p* < 0.01, *** *p* < 0.001 vs. DMSO treatment in basal condition. ^§§^ *p* < 0.01, vs. vitamin D treatment in basal condition. ^##^ *p* < 0.01, ^###^ *p* < 0.001 vs. DMSO + IL-1β treatment. *FF* genotype *n* = 3, *Ff* genotype *n* = 7. Data are represented as mean ± SD.

In the presence of IL-1β, the expression of *CYP24* was strongly upregulated in both genotypes (*p* < 0.05 for *FF* cells and *p* < 0.01 for *Ff* cells) (Figure 6A), whereas inflammation provoked an upregulation of *VDR* expression in *Ff* cells (*p* < 0.05) (Figure 6B).

### 2.3. Vitamin D Supplementation Did Not Influence the Release of IL-1β in Disc Cells

At basal level, no IL-1β was detected in the supernatant either in DMSO or 1,25(OH)_2_D_3_-treated cells. After 48 h of 1000 pg/mL IL-1β administration, an average amount of 350 ± 190 pg/mL was detected in cells, regardless of genotype or vitamin D treatment. The spontaneous degradation of IL-1β was also evaluated in culture media in the absence of cells; an average of 148.7 ± 37.4 pg/mL was found after 48 h of IL-1β administration.

### 2.4. Vitamin D Upregulated IL-1Ra Release in Basal Condition and Downregulated IL-6 Release in Inflamed Condition in Cells Bearing the FokI VDR Ff Genotype

The treatment with 1,25(OH)_2_D_3_ significantly upregulated the release of IL-1Ra only in cells bearing the *Ff* genotype in basal culture condition (*p* < 0.01) (Figure 3A). It should be noted that disc cells bearing the *Ff* genotype showed higher basal IL-1Ra concentrations—about 60 pg/mL—in comparison to the ones bearing the *FF* genotype, which had about 40 pg/mL. In inflamed conditions, 1,25(OH)_2_D_3_ showed to be able to downregulate the production of IL-6 only in the presence of a *VDR* FokI *Ff* genotype (*p* < 0.001) (Figure 3B). 

### 2.5. In Inflamed Condition Vitamin D Upregulated the Expression of ACAN in Ff Disc Cells, But Did Not Have an Effect on SOX9 and COL1A1 Expression

In all conditions, with the exception of the *Ff* cells treated with 1,25(OH)_2_D_3_ in basal medium, a decrease in *ACAN* expression was observed. Interestingly, in the presence of IL-1β, vitamin D counteracted this effect by increasing the *ACAN* expression in *Ff* genotype cells, compared to DMSO in the same inflamed condition (*p* < 0.05) (Figure 3A). 

In both basal and inflamed conditions, vitamin D did not influence the expression of *COLA1A1* and *SOX9* in cells bearing both the *FF* and *Ff* genotypes (Figure 3B,C). 

### 2.6. Vitamin D Was Able to Affect the Expression of MMPs in Cells Bearing the Ff Genotype in Both Basal and Inflamed Conditions

Vitamin D was able to increase the *MMP1*, *MMP3* and *MMP13* expression in basal condition in cells bearing the *Ff* genotype. In contrast, 1,25(OH)_2_D_3_ in an inflamed condition downregulated the expression of *MMP3* in *FF* bearing cells (*p* < 0.01), and of *MMP13* in *Ff* bearing cells (*p* < 0.001) (Figure 5A–C). 

### 2.7. Vitamin D Upregulated the Vitamin D-Dependent Signaling Pathway, but This Was Downregulated in Inflamed Cells Bearing the Ff Genotype

In general, independently from the FokI genotypes, 1,25(OH)_2_D_3_ upregulated *CYP24* expression, both in basal and inflamed conditions (*FF* genotype, *p* < 0.01, for both basal and inflamed conditions; *Ff* genotype, *p* < 0.001, for both basal and inflamed conditions) (Figure 6A). Interestingly, *CYP24* expression levels were downregulated by vitamin D in an inflamed environment in comparison with its levels in the presence of vitamin D alone, only in *Ff* bearing cells (*p* < 0.01).

Vitamin D did not affect *VDR* expression in either of the conditions, regardless of the cell genotype (Figure 6B). 

## 3. Discussion

The main finding of this study is that the cells bearing the two different FokI *VDR* gene variants show some differences in response to the treatment with vitamin D, where the *Ff* genotype was the most responsive. 

Regardless of the FokI *VDR* genotype, vitamin D had an inhibitory effect on the proliferation and metabolic activity of the disc cells while particularly favoring a pro-apoptotic induction on these kinds of cells, as already reported in previously published studies [1,2].

When the pro-inflammatory stimulus IL-1β, a cytokine well-known to induce an inflammatory and catabolic environment in the intervertebral disc [17,19,20,21] was added to the culture, the disc cells showed a similar IL-1β metabolism, regardless of genotype and vitamin D supplementation. Since a substantial amount of IL-1β is lost in the culture medium in absence of cells at the end of 48 h of incubation, the higher value detected in the cell culture after IL-1β administration suggests an active IL-1β cell secretion.

The disc cells bearing the FokI *VDR Ff* genotype were the most responsive to vitamin D, and showed an anti-inflammatory attitude by counteracting the upregulation induced by IL-1β of the pro-inflammatory IL-6, by upregulating the anti-inflammatory IL-1Ra release in basal condition and also by showing higher basal IL-1Ra concentrations in comparison with cells bearing the *FF* genotype, regardless of the vitamin D treatment. 

Moreover, in *Ff*-bearing disc cells, vitamin D supplementation provoked an upregulation of the expression of *ACAN*, a marker of disc phenotype expressed in terminally differentiated cells, counteracting the strong downregulation of this gene observed after IL-1β treatment in absence of vitamin D. The downregulation of *ACAN* observed in basal condition after vitamin D treatment in cells bearing the *FF* genotype was in accordance with what was previously observed [2], and suggested a different behavior of the cells in response to the vitamin D treatment for what concerned the aggrecan production, depending on their genotype. In particular, the *Ff* bearing cells were, even in this case, most responsive to vitamin D in terms of preservation of the expression of this crucial marker of phenotype.

Even for what concerned the catabolic response, *Ff*-bearing cells were the most responsive, showing an upregulation of the MMP’s expression of vitamin D mediated in basal condition. 

On the contrary, in an inflamed environment, 1,25(OH)_2_D_3_ slightly downregulated the expression of *MMP3* in *FF* bearing cells and of *MMP13* in *Ff* bearing cells, thus counteracting the increase of these MMP’s expression mediated by IL-1β. 

Regardless of genotype, the expression of *CYP24* was strongly upregulated by both IL-1β and 1,25(OH)_2_D_3_ (even more strongly), thus showing that both stimuli influenced the vitamin D signaling pathway. Only in *Ff* bearing cells were the levels of this marker slightly downregulated by vitamin D in an inflamed environment with respect to the basal condition, making it likely to counteract the inflammation-mediated catabolic effects. A further confirmation of the influence of inflammation on the vitamin D signaling pathway was shown by the modulation of *VDR* expression. Only in *Ff* bearing cells was a slight increase of the expression of this gene observed in an inflamed condition. As already published [2], vitamin D did not affect the expression of this receptor.

The main limitation of this study is the absence of data regarding cells bearing the *ff* genotype, due to the low frequencies of this genotype in the Italian population (about 13%), as already published [7]. Moreover, the relevant data obtained at transcriptional level should also be confirmed at protein level, and further evaluation of subchondral bone cells obtained from vertebral bodies should be performed in view to confirm the anti-proliferative and catabolic effects of vitamin D observed in this study, in order to assess the potential effects of vitamin D on bone metabolism in the presence of specific functional FokI *VDR* genotypes. 

These pieces of evidence suggest that calcitriol, at the pharmacological concentration used in this study, has a general anti-proliferative and catabolic effect on disc cells. For these reasons, the use of vitamin D to treat the degenerated disc with homeostatic or regenerative purposes is not suggestable, particularly for patients bearing *FF VDR* genotype which showed the highest risk of developing degenerative disc diseases [7,8,12]. Nevertheless, cells bearing the *Ff* genotype were the most responsive to the vitamin D supplementation, showing anti-inflammatory and catabolic behaviors in response to the treatment with the hormone, likely related to matrix remodeling. Starting from these pieces of evidence, the systemic or local supplementation of vitamin D could be considered as a treatment option for a particular subgroup of patients presenting disc degeneration related to osteochondrosis. In fact, this latest pathological condition involves not only the fibro-cartilaginous disc, but also the upper and lower bony-cartilaginous endplates, limiting the discs. In particular, the subchondral bone of these patients shows typical degenerative features, such as a multilevel presence of sclerosis and Schmorl’s nodes [22,23]. Predisposing factors for spinal osteochondrosis are the presence of bone metabolic diseases, including osteomalacia, hyperparathyroidism, Paget’s disease, infections, neoplasm and osteoporosis, which may weaken the vertebral bodies and allow Schmorl’s nodes to form [24]. Although the endplates serve as the main route of nutrient supply into the disc [25], their calcification, due to the presence of chronic lesions, may lead to the loss of this role and may contribute to disc degeneration in this pathology. Also, the immune system has been postulated as having a role in the development of osteochondrosis. Discs that herniated into the vertebral endplate and eventually into the bone marrow could be recognized as foreign material, leading to an immune reaction with inflammatory cell infiltration, edema, influx of cytokines, and pain. Moreover, the herniation of the nucleus pulposus into the vertebrae can determine a cross-talk between a dysregulated immune system and bone metabolism, resulting in an imbalance of bone remodeling and consequently leading to bone loss [26]. This is a vicious circle where bone loss may predispose affected vertebrae to herniation of more disc material, exacerbating the condition. 

In view of the importance of vitamin D in skeletal homeostasis [27] and of its immunomodulatory properties [16], and due to the peculiar pathological features of osteochondrosis, a vitamin D supplementation may be suggested to treat the subchondral sclerosis and the degenerated vertebral bone of the patients with spinal osteochondrosis bearing the *Ff* genotype, which showed to be the most promising subject concerning their responsiveness to the vitamin D treatment.

## 4. Materials and Methods

### 4.1. Study Population and Tissue Samples Collected

The study was approved by the ethics committee of the San Raffaele hospital (Protocol GenVDisc Version 1, 20 November 2015) and specimens were collected with patient-informed consent. Waste material from the lumbar intervertebral disc of 15 patients with a mean age of 54.6 ± 13.2 and who were affected by spine disorders was collected during discectomy. Demographic features, disc level, and patients’ genotypes are listed in Table 1.

### 4.2. Isolation and Expansion of Disc Cells

Disc cells were isolated by enzymatic digestion (37 °C, 22 h) using type II collagenase (Worthington Biochemical Co., Lakewood, NJ, USA) at the concentrations of 224 U/mL for NP, 560 U/mL for AF and 336 U/mL for CEP samples [28]. After digestion, the samples were filtered through a cell strainer and centrifuged (1000× *g*, 5 min). The cells were counted and plated at 10^5^ cells/cm^2^, (37 °C, 5% CO_2_) in 1 mg/mL of low-glucose Dulbecco’s-modified Eagle medium (LG-DMEM, Thermo Fisher Scientific, Waltham, MA, USA) supplemented with 10% fetal bovine serum (FBS, Lonza, Basel, Switzerland), 0.29 mg/mL l-glutamine, 100 U/mL penicillin, 100 µg/mL streptomycin, 10 mM Hepes, 1 mM sodium pyruvate (all reagents from Thermo Fisher Scientific). During culture, the medium was replaced twice a week. At confluence, cells were detached using 0.05% trypsin/0.053 mM EDTA (Thermo Fisher Scientific) and plated at 5 × 10^3^ cells/cm^2^ for the following passages. The cells were expanded up to passage 3 and then used for the experiments.

### 4.3. Determination of FokI VDR Genotypes

Genomic DNA was extracted from disc cells according to the procedure of the Pure Link^TM^ Genomic DNA Mini kit (Invitrogen, Carlsbad, CA, USA) and quantified spectrophotometrically (NanoDrop, Thermo Fisher Scientific). FokI *VDR* genotypes were determined by using TaqMan SNP Genotyping Assay (Thermo Fisher Scientific) for rs2228570 polymorphism and a StepOne Plus instrument (Thermo Fisher Scientific).

### 4.4. Calcitriol (1,25(OH)_2_D_3_) and IL-1β Treatment Protocols

Monolayer cultured cells were allowed to attach for 24 h in standard culture medium. 

For cell cycle and apoptosis analysis, the cells were cultured for 24 h in low serum medium (5% FBS) to decrease the vitamin D binding protein contained in a serum, which might have interfered with the experiment. 10^−8^ M 1,25(OH)_2_D_3,_ or vehicle (0.1% DMSO) (Sigma-Aldrich, St. Louis, MO, USA), were then added, and the medium changed at day 3 when the vitamin D treatment was repeated; the cells were collected after 6 days of treatment for the analysis of the cell cycle and apoptosis.

For the evaluation of the response to inflammation, 10^−8^ M 1,25(OH)_2_D_3,_ or vehicle (0.1% DMSO), was added to 80% confluent cells in 5% FBS in the presence or absence of 1 ng/mL IL-1β (Sigma-Aldrich). The cells and supernatant were then evaluated after 48 h of treatment [29].

### 4.5. Cell Cycle Analysis

Cell cycle progression was evaluated by monitoring the DNA content through the Tali^®^ Cell Cycle Kit (Thermo Fisher Scientific) consisting of an all-in-one solution containing propidium iodide, RNase A, and Triton X-100 to label cells. After 6 days of 1,25(OH)_2_D_3_ or DMSO treatment, the cells were detached using 0.05% trypsin/0.053 mM EDTA, centrifuged at 500× *g* for 5 min, washed with phosphate buffered saline solution (PBS), centrifuged again at the same previous conditions, transferred to ice and fixed into a single cell suspension with ice-cold 70% ethanol in distilled water. Later on, the cells were placed at −20 °C overnight, then centrifuged at 1000× *g* for 5 min at 4 °C to remove the ethanol and washed in PBS. After a centrifugation step at 500× *g* for 10 min at 4 °C, the PBS was removed and cells were resuspended in 200 µL of Tali^®^ Cell Cycle Solution at RT for 30 min in the dark. The cells were briefly vortexed to gently resuspend them before the cell cycle analysis using the Tali^®^ Image-Based Cytometer (Thermo Fisher Scientific). Using the instrument software, small cells (indicating debris) and large cells (indicating aggregates) were gated out of the analysis by setting the gate on the cell size; the threshold gates for each cell cycle phase were also set.

### 4.6. Evaluation of Apoptosis

Tali^®^ Apoptosis Assay Kit–Annexin V Alexa Fluor^®^ 488 and Propidium Iodide (Thermo Fisher Scientific) were used to evaluate the presence of apoptosis after the 1,25(OH)_2_D_3_ or DMSO treatment. 

Briefly, the cells were harvested and centrifuged to discard the supernatant. Annexin binding buffer and Annexin V Alexa Fluor^®^ 488 were added to the cells and the mixture was left at RT in the dark for 20 min. The cells were centrifuged and resuspended in Annexin binding buffer added to Tali^®^ Propidium Iodide. Samples were incubated at RT in the dark for 5 min. The stained cells were then loaded onto the Tali^®^ Image-Based Cytometer. Apoptotic cells showed green fluorescence, necrotic cells showed red fluorescence, dead cells (apoptosis/secondary necrosis) showed yellow fluorescence, and live cells showed little to no fluorescence.

### 4.7. Gene Expression Analysis

Total RNA was isolated from cell lysates using the PureLink^®^ RNA Mini Kit (Thermo Fisher Scientific) and quantified spectrophotometrically (NanoDrop). 

RNA were reverse-transcribed to cDNA employing the iScript cDNA Synthesis Kit (Bio-Rad Laboratories, Hercules, CA, USA). Gene expression was evaluated by real-time PCR (StepOne Plus instrument). cDNA was incubated with a PCR mixture, including the TaqMan^®^ Gene Expression Master Mix and TaqMan^®^ Gene Expression Assays (Thermo Fisher Scientific). 

The expression of phenotype markers such as *ACAN*, Hs00153936_m1, *COL1A1*, Hs01076777_m1, *SOX9*, Hs00165814_m1, vitamin D-related genes such as *CYP24*, Hs00167999_m1, *VDR*, Hs01045840_m1, matrix metalloproteases *MMP1*, Hs00899658_m1, *MMP3*, Hs00968305_m1 and *MMP13*, Hs00233992_m1 was analyzed after IL-1β treatment.

The previously-validated *TBP*, Hs00427620_m1 was used as a housekeeping gene [14]. Data was expressed according to the dCt method. 

### 4.8. Determination of Cytokines

Concentrations of soluble IL-1β, IL-1Ra and IL-6 in cell culture medium after 48 h of treatment with 1,25(OH)_2_D_3_ or DMSO in the presence or absence of IL-1β were determined by commercially-available ELISA according to the manufacturers’ instructions (PeproTech, Rocky Hill, NJ, USA). 

The detection ranges were: 23–1500 pg/mL for IL-1β, 24–1500 pg/mL for IL-6 and 23–1500 pg/mL for IL-1Ra.

### 4.9. Statistical Analysis

Statistical analysis was performed using GraphPad Prism v5.0 software (GraphPad Software Inc., La Jolla, CA, USA). All values are expressed as the mean ± SD. Normal distribution of values were assayed by the Kolmogorov-Smirnov normality test. Paired comparisons were performed by using a two-tailed *t* test. In the case of not normally distributed values, repeated measures were compared with the Kruskal-Wallis test with the Dunns’ correction. The significance level was set at a *p*-value lower than 0.05.

## Figures and Tables

**Table 1:** Demographic features, disc level and genotypes of the recruited patients.

Sex	Age	Spine Disorder	Disc Level	FokI *VDR* Genotype
F	27	Spondylolisthesis	L5-S1	*FF*
F	39	Discopathy	L4-L5	*FF*
F	46	Degenerative discopathy	L5-S1	*Ff*
F	48	Spondylolisthesis	L5-S1	*FF*
F	48	Degenerative discopathy	L5-S1	*Ff*
F	50	Discopathy	L5-S1	*Ff*
F	50	Discopathy	L5-S1	*Ff*
M	52	Discopathy	L5-S1	*Ff*
F	55	Discopathy	L5-S1	*Ff*
M	57	Discopathy	L5-S1	*Ff*
F	65	Discopathy	L5-S1	*Ff*
F	66	Degenerative discopathy	L5-S1	*FF*
F	66	Discopathy	L5-S1	*Ff*
F	74	Discopathy	L5-S1	*Ff*
F	76	Discopathy	L5-S1	*Ff*

## Abbreviations

VDR	Vitamin D receptor
IL-1Ra	Interleukin-1 receptor antagonist
IL-6	Interleukin 6
NP	Nucleus pulposus
AF	Annulus fibrosus
CEP	Osteo-cartilaginous endplate
IL-1β	Interleukin 1β
ACAN	Aggrecan
COL1A1	Collagen Type I Alpha 1 Chain
SOX9	SRY-Box 9
MMP	Matrix metalloprotease
CYP24	Cytochrome P450 Family 24 Subfamily A Member 1
DMSO	Dimethyl sulfoxide
TBP	TATA-binding protein

## References

[1] Gruber H.E., Hoelscher G., Ingram J.A., Chow Y., Loeffler B., Hanley E.N. (2008). 1,25(OH)_2_-vitamin D3 inhibits proliferation and decreases production of monocyte chemoattractant protein-1, thrombopoietin, VEGF, and angiogenin by human annulus cells in vitro. Spine.

[2] Colombini A., Lanteri P., Lombardi G., Grasso D., Recordati C., Lovi A., Banfi G., Bassani R., Brayda-Bruno M. (2012). Metabolic effects of vitamin D active metabolites in monolayer and micromass cultures of nucleus pulposus and annulus fibrosus cells isolated from human intervertebral disc. Int. J. Biochem. Cell Boil..

[3] Colombini A., Cauci S., Lombardi G., Lanteri P., Croiset S., Brayda-Bruno M., Banfi G. (2013). Relationship between vitamin D receptor gene (VDR) polymorphisms, vitamin D status, osteoarthritis and intervertebral disc degeneration. J. Steroid Biochem. Mol. Boil..

[4] Chen L., Zhao S., Niu F., Bi G.B. (2017). Association between vitamin D receptor gene polymorphisms and intervertebral disc degeneration: A meta-analysis. J. Orthop. Sci..

[5] Jiang H., Qin Z.L., Zong S.H., He M.L., Zhan X.L., Xiao Z.M., Wei Q.J. (2017). Vitamin D receptor gene polymorphisms and lumbar disc degeneration: A systematic review and meta-analysis. Eur. Spine J..

[6] Zhao J., Yang M., Shao J., Bai Y., Li M. (2015). Association between VDR FokI Polymorphism and Intervertebral Disk Degeneration. Genom. Proteom. Bioinform..

[7] Colombini A., Brayda-Bruno M., Lombardi G., Croiset S.J., Vrech V., Maione V., Banfi G., Cauci S. (2014). FokI polymorphism in the vitamin D receptor gene (VDR) and its association with lumbar spine pathologies in the Italian population: A case-control study. PLoS ONE.

[8] Colombini A., Brayda-Bruno M., Ferino L., Lombardi G., Maione V., Banfi G., Cauci S. (2015). Gender differences in the VDR-FokI polymorphism and conventional non-genetic risk factors in association with lumbar spine pathologies in an Italian case-control study. Int. J. Mol. Sci..

[9] Colombini A., Brayda-Bruno M., Lombardi G., Croiset S.J., Ceriani C., Buligan C., Barbina M., Banfi G., Cauci S. (2016). BsmI, ApaI and TaqI Polymorphisms in the Vitamin D Receptor Gene (VDR) and Association with Lumbar Spine Pathologies: An Italian Case-Control Study. PLoS ONE.

[10] Mashayekhi S., Saberi A., Salehi Z., Biazar G., Mehrdel R. (2018). VDR and GC gene polymorphisms modulate the risk of lumbar disc degeneration in Iran. Clin. Neurol. Neurosurg..

[11] Brayda-Bruno M., Vigano M., Cauci S., Vitale J.A., de Girolamo L., De Luca P., Lombardi G., Banfi G., Colombini A. (2017). Plasma vitamin D and osteo-cartilaginous markers in Italian males affected by intervertebral disc degeneration: Focus on seasonal and pathological trend of type II collagen degradation. Clin. Chim. Acta Int. J. Clin. Chem..

[12] Cauci S., Vigano M., de Girolamo L., De Luca P., Perucca Orfei C., Banfi G., Lombardi G., Brayda-Bruno M., Colombini A. (2017). High Levels of Circulating Type II Collagen Degradation Marker (CTx-II) Are Associated with Specific VDR Polymorphisms in Patients with Adult Vertebral Osteochondrosis. Int. J. Mol. Sci..

[13] Uitterlinden A.G., Fang Y., Van Meurs J.B., Pols H.A., Van Leeuwen J.P. (2004). Genetics and biology of vitamin D receptor polymorphisms. Gene.

[14] Costanzo P., Santini A., Fattore L., Novellino E., Ritieni A. (2015). Toxicity of aflatoxin B1 towards the vitamin D receptor (VDR). Food Chem. Toxicol. Int. J. Publ. Br. Ind. Biol. Res. Assoc..

[15] Jurutka P.W., Whitfield G.K., Hsieh J.C., Thompson P.D., Haussler C.A., Haussler M.R. (2001). Molecular nature of the vitamin D receptor and its role in regulation of gene expression. Rev. Endocr. Metab. Disord..

[16] Guillot X., Semerano L., Saidenberg-Kermanac’h N., Falgarone G., Boissier M.C. (2010). Vitamin D and inflammation. Joint Bone Spine.

[17] Molinos M., Almeida C.R., Caldeira J., Cunha C., Goncalves R.M., Barbosa M.A. (2015). Inflammation in intervertebral disc degeneration and regeneration. J. R. Soc. Interface.

[18] Colombini A., Lombardi G., Corsi M.M., Banfi G. (2008). Pathophysiology of the human intervertebral disc. Int. J. Biochem. Cell Boil..

[19] Le Maitre C.L., Freemont A.J., Hoyland J.A. (2005). The role of interleukin-1 in the pathogenesis of human intervertebral disc degeneration. Arth. Res. Ther..

[20] Le Maitre C.L., Pockert A., Buttle D.J., Freemont A.J., Hoyland J.A. (2007). Matrix synthesis and degradation in human intervertebral disc degeneration. Biochem. Soc. Trans..

[21] Johnson Z.I., Schoepflin Z.R., Choi H., Shapiro I.M., Risbud M.V. (2015). Disc in flames: Roles of TNF-alpha and IL-1beta in intervertebral disc degeneration. Eur. Cells Mater..

[22] McFadden K.D., Taylor J.R. (1989). End-plate lesions of the lumbar spine. Spine.

[23] Aufdermaur M. (1981). Juvenile kyphosis (Scheuermann’s disease): Radiography, histology, and pathogenesis. Clin. Orthop. Relat. Res..

[24] Kyere K.A., Than K.D., Wang A.C., Rahman S.U., Valdivia-Valdivia J.M., La Marca F., Park P. (2012). Schmorl’s nodes. Eur. Spine J..

[25] Urban J.P., Smith S., Fairbank J.C. (2004). Nutrition of the intervertebral disc. Spine.

[26] Zhang N., Li F.C., Huang Y.J., Teng C., Chen W.S. (2010). Possible key role of immune system in Schmorl’s nodes. Med. Hypotheses.

[27] Goltzman D. (2018). Functions of vitamin D in bone. Histochem. Cell Biol..

[28] Lopa S., Ceriani C., Cecchinato R., Zagra L., Moretti M., Colombini A. (2016). Stability of housekeeping genes in human intervertebral disc, endplate and articular cartilage cells in multiple conditions for reliable transcriptional analysis. Eur. Cells Mater..

[29] Kim J.H., Choi H., Suh M.J., Shin J.H., Hwang M.H., Lee H.M. (2013). Effect of biphasic electrical current stimulation on IL-1beta-stimulated annulus fibrosus cells using in vitro microcurrent generating chamber system. Spine.

